# Mitochondrial point heteroplasmy: insights from deep-sequencing of human replicate samples

**DOI:** 10.1186/s12864-024-09963-z

**Published:** 2024-01-10

**Authors:** Marina Korolija, Viktorija Sukser, Kristian Vlahoviček

**Affiliations:** 1 Biology and Fibres Department, Forensic Science Centre “Ivan Vučetić”, Ministry of the Interior of the Republic of Croatia, Ilica 335, HR-10000 Zagreb, Croatia; 2 Bioinformatics group, Division of Molecular Biology, Department of Biology, Faculty of Science, University of Zagreb, Horvatovac 102a, HR-10000 Zagreb, Croatia

**Keywords:** Mitochondrial point heteroplasmy (PHP), Minor allele frequency (MAF), PHP landscape

## Abstract

**Background:**

Human mitochondrial heteroplasmy is an extensively investigated phenomenon in the context of medical diagnostics, forensic identification and molecular evolution. However, technical limitations of high-throughput sequencing hinder reliable determination of point heteroplasmies (PHPs) with minor allele frequencies (MAFs) within the noise threshold.

**Results:**

To investigate the PHP landscape at an MAF threshold down to 0.1%, we sequenced whole mitochondrial genomes at approximately 7.700x coverage, in multiple technical and biological replicates of longitudinal blood and buccal swab samples from 11 human donors (159 libraries in total). The results obtained by two independent sequencing platforms and bioinformatics pipelines indicate distinctive PHP patterns below and above the 1% MAF cut-off. We found a high inter-individual prevalence of low-level PHPs (MAF < 1%) at polymorphic positions of the mitochondrial DNA control region (CR), their tissue preference, and a tissue-specific minor allele linkage. We also established the position-dependent potential of minor allele expansion in PHPs, and short-term PHP instability in a mitotically active tissue. We demonstrate that the increase in sensitivity of PHP detection to minor allele frequencies below 1% within a robust experimental and analytical pipeline, provides new information with potential applicative value.

**Conclusions:**

Our findings reliably show different mutational loads between tissues at sub-1% allele frequencies, which may serve as an informative medical biomarker of time-dependent, tissue-specific mutational burden, or help discriminate forensically relevant tissues in a single person, close maternal relatives or unrelated individuals of similar phylogenetic background.

**Supplementary Information:**

The online version contains supplementary material available at 10.1186/s12864-024-09963-z.

## Background

The mitochondrial (mt) genome represents a small yet extremely important fraction of human genetic material. Its ubiquitously expressed genes involved in ATP production are essential for the survival of virtually all human cells. Distinctive biological features of mtDNA are ascribable to its physical detachment from the nuclear (n) genome and nucleus-independent replication, which produces multiple circular mtDNA copies per cell. Cytoplasmic localization and haploid nature provide additional specific characteristics of the mt genome, such as matrilineal inheritance and a presumable lack of recombination. This renders mutations as predominant drivers of mitochondrial haplotype diversity [1, 2]. As such, mtDNA holds a prominent position in forensic, medical and anthropological genetics.

The emergence of massively parallel sequencing (MPS) enabled simultaneous, targeted, high-coverage resequencing of entire mitochondrial genomes from many samples on a routine basis [3–14]. The MPS approach brought added value to traditional Sanger-type sequencing, which is typically restricted to approximately 1.1 kbp of the noncoding control region (CR), by allowing cost-effective access to the information stored in approximately 15.5 kbp of the transcribed region (TR). Another improvement of MPS compared to previous sequencing techniques is genome-wide analysis of alternative sequence variants with a significantly lower detection threshold [8]. The rapid increase in high-quality mtDNA data started to provide more comprehensive insight into mtDNA diversity within populations, across tissues and between maternal relatives. Novel findings, combined with optimized experimental and bioinformatics pipelines, contribute to reassessment of the role and relevance of mtDNA across various fields of genetics [8, 15–18].

Mitochondrial point heteroplasmy (PHP) manifests as single base differing populations of mtDNA molecules coexisting within a cell, a tissue, across several tissues or the entire body of an individual [2]. The quantity, distribution and tissue specificity of PHPs in the human mt genome are well characterized, especially with the use of MPS. However, there are still considerable knowledge gaps on the low-level PHP landscape, which overlaps with sequencing noise and differs from the landscape shaped by more strongly expanded mutations in both germline and somatic tissues [4, 5, 10, 19–21]. Consequently, a low-level PHP pattern cannot be inferred but instead should be established experimentally in a variety of tissues, haplotype backgrounds and life stages. Although sophisticated techniques for detecting low-frequency genetic variants do exist [22, 23], they are still too uncommon to allow thorough assessment and potential consideration of low-frequency changes in mtDNA as new molecular markers. Applicative potential of low-level PHP patterns appears particularly high in forensics because of its capacities in discrimination of unrelated individuals with identical haplotypes, discrimination of close maternal relatives, and determination of age, tissue type and/or environmental influences [8, 24]. However, to date, the majority of studies addressing PHP patterns with minor allele frequency (MAF) below threshold of 1% were performed in clinical context by the analysis of medically relevant tissues [3–6, 10, 25–27].

The difficulty of accurate low-level PHP calling is primarily imposed by technical limitations of sequencing and a number of factors contributing to artefact generation throughout the sequencing workflow [22, 28–31]. Additionally, the results are often questioned because of deficient methodology for discriminating true from false positives, discrepancies from known mtDNA genetics, and confounding signals from nuclear mitochondrial DNA segments (NUMTs) [8, 32–35]. Determination of the low-level PHP landscape usually relies on single-pass sequenced libraries, accompanied by more or less successful post-sequencing error mitigation. Replicate sequencing as an experimental approach for reducing sequencing errors is still rarely performed in MPS experiments because it can substantially increase the cost of the study, especially in the case of large sample pools [36]. Moreover, sequencing workflows in general do not undergo thorough validation prior to deployment, which hinders understanding the nature of workflow-specific errors [29, 34]. It is therefore recommended that validation of individual laboratory setups should precede any PHP analysis. Sequencing experiments should be performed at least in duplicate and preferably analysed by independent data analysis pipelines to exclude pipeline-specific artefacts [28, 35].

In this work, we reanalyse at a 0.1% MAF threshold the blood and buccal swab samples and two cell-line controls, which were repeatedly sequenced as technical replicates in our previous validation study [37]. In addition to mapping the PHP landscape, we also sequenced and analysed biological replicates taken in time intervals to examine PHP stability in mitotically active tissues. We hypothesize that replicate sequencing and the extensive validation of the sequencing workflow enable clearer insight into the low-level PHP pattern. In our dataset, by applying strict technical criteria, we repeatedly, reproducibly and concordantly detected a number of minor alleles by two independent data analysis pipelines. We consider all described heteroplasmic positions and allele frequencies from a biological perspective and place them in the context of similar published datasets [6, 10]. We present arguments in favour of characterizing reported PHPs as genuine, but we also assess the risk of identifying false positives as authentic minor alleles. The rationale underlying this approach is to advance the investigation of subtle mutational changes in mtDNA, with the aim of enhancing their potential for the development of novel forensic and/or clinical markers.

## Methods

To investigate the PHP landscape at an MAF threshold down to 0.1%, we sequenced whole mitochondrial genomes in multiple technical and biological replicates of longitudinal blood and buccal swab samples from 11 human donors (159 libraries in total).

### Technical and biological replicate samples

All sequencing data originate from our previous MiSeq FGxTM mtDNA workflow evaluation study carried out through eight sequencing runs at an average sample sequencing depth of 7.746 reads per position [37] were reanalysed by applying the 0.1% MAF threshold. The study included sequencing of blood and buccal swab samples from 11 donors and the Standard Reference Material® (SRM®) 2392 Component #1 CHR (abbreviated as SRM-C) and SRM® 2392-I HL-60 (abbreviated as SRM-H) from the National Institute of Science and Technology (NIST) (Gaithersburg, MD, USA) [38, 39] (validation study). Additionally, buccal swab and blood samples from 9 out of 11 participants from the validation study were taken in time intervals for the assessment of temporal stability of PHP distribution and magnitude (longitudinal study). Sequencing results from the longitudinal study, carried out through three sequencing runs at an average sample depth of 7,751 reads per position, were also analysed by applying a 0.1% MAF threshold. Library preparation and sequencing for both validation and longitudinal studies were performed identically, as described in [37] and briefly overviewed in SI Appendix, Methods. The samples and the plan of both validation and longitudinal study sequencing experiments are outlined in Dataset S1, and the plan of the longitudinal study is additionally described in SI Appendix, Methods. Haplogroup assignment was performed based on full haplotypes of all study participants [37] by using HaploGrep2 v.2.1.1 [40]. Computationally assigned haplogroups were manually validated against the PhyloTree, Build 17 [41] and are given in Dataset S1.

### Heteroplasmy-specific analyses

In contrast to the internally established threshold for forensics-safe PHP reporting [37], this work uses less stringent criteria set in BaseSpace® mtDNA Variant Processor v1.0.0 App, with analysis threshold (AT) = 0.1%, interpretation threshold (IT) = 0.1%, and minimum read count = 10. In case of inability to detect minor alleles in one or more replicates (either technical or biological) of a particular person/tissue combination, most commonly caused by insufficient read depth at certain positions, the minimum read count was lowered to 2 reads, and the replicate in question was reanalysed. All results underwent visual inspection in BaseSpace® mtDNA Variant Analyzer v1.0.0 App and were exported to Excel for detailed review. The BaseSpace® mtDNA data analysis workflow is described in detail in [37] and briefly in SI Appendix, Methods.

For low-level PHP confirmation, only those PHPs were considered that were completely reproducible within a single person/tissue combination and detectable by MiSeq FGxTM-sequenced reads from both strands across all biological, PCR and library replicates (total of 159 libraries from blood and buccal swabs of 11 donors and eight libraries from two SRM samples). Of those, only heteroplasmic positions exceeding the previously established GQ (genotype quality) score threshold of 41 [37] were further reviewed, thereby excluding as stochastic errors the majority of heteroplasmic positions randomly appearing throughout the replicates of different samples. PHPs that met these criteria were further filtered by checking concordance with the results of the independent run on the NextSeq®500. Finally, concordant PHPs were analysed in parallel with an in-house bioinformatics pipeline as described in SI Appendix, Methods.

Additional criteria for PHP confirmation, patterns of erroneous PHP calls and information on sequencing of biological replicates for longitudinal study are described in SI Appendix, Methods. Single allele frequencies at all identified PHP positions confirmed by both bioinformatics approaches are given per sample per tissue in Datasets S2-S16. Allele frequencies expressed as single, average or median MAF values obtained with the BaseSpace® mtDNA data analysis workflow are given in Dataset S17, Tables 1 and 2 and throughout the manuscript. In subsequent text, we will refer to “low-level” PHPs as those with MAF < 1% (explanation in SI Appendix, Methods).

**Table 1:** Control region point heteroplasmies detected in more than one person

Mitochondrial (MT) locus	Position	Major allele	Minor allele	Substitution rate [46]	No. Persons	Total no. PHPs (both tissues)	Median MAF^*^% (MIN% -MAX%)	No. persons reported in [10]	No. mother-child duos reported in [6]
MT-HV2	189	A	G	31	6	7	0.5 (0.3–0.6)	103	3
MT-HV1	16129	G	A	86	6	6	0.2 (0.2–0.6)	1 (5 A>G)^‡^	/
MT-HV2	152	T	C	157	5	7	0.2 (0.2–5.1)	10	1
MT-HV1	16293	A	G	17	5	5	0.3 (0.2–0.5)	1	/
Control region	16519	C	T	209	4	6	0.3 (0.1–2.8)	1 (9 T>C)^‡^	/
MT-HV2	146	T	C	109	4	4	0.3 (0.1–0.4)	2	/
MT-HV1	16093	C	T	79	3	6	3.0 (1.6–12)	6	3
MT-HV2	204	T	C	43	3	5	0.4 (0.3–2.1)	26	1
MT-HV1	16286	T	C	5	2	4	1.9 (0.9–39.6)	/	/
MT-HV1	16311	T	C	120	2	3	0.5 (0.3–6.8)	/ (2 C>T)^‡^	/
MT-HV2	214	A	G	10	2	3	2.3 (0.2–49.6^†^)	/ (2 A>T)	3
Control region	16390	G	A	31	2	3	2.4 (0.1–18.3)	17	/
MT-HV2	195	T	C	98	2	2	2.6 (0.4–4.7)	3	1

^*^ median MAF value was calculated from all MAFs detected at designated positions across buccal and blood samples of 11 individuals

^†^ minor allele A

^‡^ in brackets, number of persons with point heteroplasmy of reverse substitution direction (i.e., where major and minor allele nucleotides are reversed)

**Table 2:** Point heteroplasmies (PHPs) detected within the mtDNA coding region in 9 out of total 11 individuals. Two individuals’ samples do not exhibit PHPs in the coding region

Age at sampling	Individual	Position	Substitution rate [46]	Mitochondrial (MT) locus [45]	Mutation*	Major allele	Minor allele	MAF Buccal	MAF Blood	PolyPhen-2 prediction [50]
48	MW-0080	3586	/	MT-ND1	NS	C	T	3.6%	0	BENIGN
42	MW-0020	7348	/	MT-CO1	NS	T	C	0.6%	0.8%	BENIGN
38	MW-0067	8034	/	MT-CO2	NS	T	C	0.5%	0.8%	BENIGN
42	MW-0020	9325	2	MT-CO3	NS	T	C	6.3%	3.6%	BENIGN
37	MW-0073	15731	3	MT-CYB	NS	G	A	0.2%	0	BENIGN
38	MW-0026	9164	/	MT-ATP8	NS	T	C	0	0.3%	**PROBABLY DAMAGING**
42	MW-0020	9622	/	MT-CO3	NS	C	T	0	0.1%	**PROBABLY DAMAGING**
41	MW-0065	9774	/	MT-CO3	NS	G	A	0.3%	0	**PROBABLY DAMAGING**
37	MW-0012	9987	/	MT-CO3	NS	T	C	0	0.8%	**PROBABLY DAMAGING**
41	MW-0065	11634	/	MT-ND4	NS	G	A	0	0.4%	**PROBABLY DAMAGING**
41	MW-0065	14898	/	MT-CYB	NS	T	C	0.4%	0	**PROBABLY DAMAGING**
41	MW-0065	6179	3	MT-CO1	S	G	A	1.0%	3.9%	
58	MW-0087	8955	1	MT-ATP7	S	T	C	3.2%	2.0%	
38	MW-0026	9740	1	MT-CO3	S	C	T	20.7%	17.5%	
38	MW-0026	11887	3	MT-ND4	S	G	A	0	0.3%	
38	MW-0067	14020	4	MT-ND5	S	T	C	0.4%	0	
38	MW-0026	14539	/	MT-ND6	S	A	G	27.7%	25.5%	
38	MW-0026	14560	12	MT-ND6	S	G	A	0	0.4%	
37	MW-0073	15343	/	MT-CYB	S	C	T	0.1%	0	

*mutation types: S = *synonymous*; NS = *non-synonymous*

## Results

### Positive controls

Mitochondrial genotypes of SRM-C and SRM-H established by sequencing are completely concordant with NIST Certificates of analysis [38, 39]. In addition, all PHPs reported in the NIST publication [42] were also confirmed (*m.64C>Y* in SRM-C and *m.2445T>Y*, *m.5149C>Y*, and *m.1207T>Y* in SRM-H) (Datasets S2 and S3). Regardless of different sequencing platforms and bioinformatics approaches, the frequencies of minor alleles are comparable, illustrating the robustness of PHP identification by MPS [42] (Datasets S2 and S3).

By applying a minor allele calling threshold of 0.1%, we discovered two additional positions (1201 and 4929 in SRM-C) potentially affected by low-level heteroplasmy (Dataset S2). Position 1201 is identified as an *A>G* transition with an average frequency of minor allele G of 0.5%. Parameters such as minimal BaseSpace Q score of 45 in seven replicates, average depth for the position (Dataset S2), strand bias (SB) score (Dataset S2) and the absence of unusual alignment patterns as verified in IGV [43, 44] indicate true PHP. Minor allele G, detected in *m.1201A>R*, is reported in Mitomap [45] as a homoplasmic *MT-RNR1* gene variant in one sequence with a C4a haplogroup background (GenBank accession MH120737.1), and heteroplasmy *m.1201A>R* is reported in one individual among 152 analysed: in blood (MAF 8.5%), liver (MAF 0.5%) and skin (MAF 0.6%) [27]. However, although documented in vivo, the confirmative value for low-level PHP found in vitro should be considered with caution because cell culture-induced mutational changes probably differ from those occurring in vivo.

Position *m.4929* PHP is a *T>C* transition, with an average frequency of minor allele C of 0.8% (Dataset S2). Again, parameters such as minimal BaseSpace Q score of 46, average depth (Dataset S2), SB score (Dataset S2) and the absence of alignment artefacts indicate true PHP. This position is particularly interesting since the major variant T, while identified as homoplasmic by NIST [42], appears to be consistently heteroplasmic, with an approximate frequency of the major allele T of 99.2%. Variant C4929T is generally underrepresented in human or even primate individual genomes [45, 46], so it probably reflects specific cell culture conditions, as discussed later.

### Internal evaluation samples

In addition to the usage of SRMs, validation was performed by sequencing mtDNA replicates from buccal swabs and blood samples originating from two female donors, designated MW-0002 and MW-0020 (Table S1). For person MW-0002, a total of 16 replicates were sequenced for each tissue type (Datasets S4, S5). For person MW-0020, a total of 14 replicates were sequenced from buccal swabs (Dataset S6), in addition to the 12 replicates for the sample mixture study, where MW-0020 constituted the major contributor [37]. Owing to the high ratio of major contributor in the mixed sample (95.0-99.5%), all PHPs detected in the single-source replicates were detectable in the mixed sample replicates as well [37], including two low-level PHPs. Blood samples of MW-0020 were sequenced 6 times (Dataset S7).

Donor MW-0002 at age 27 years exhibited two low-level PHPs in buccal swab, one low-level and one high-level PHP in blood (Datasets S4, S5). Donor MW-0020 at age 42 exhibited two low-level and two high-level PHPs in buccal swab, nine low-level and one high-level PHP in blood (**Datasets S6, S7**). A detailed description of reported PHPs is given in SI Appendix, Results.

### Longitudinal PHP study

To gain insight into the temporal effects of mtDNA plasticity, we selected nine individuals to be sampled three times during two-year period (Dataset S1). By monitoring a total of 131 presumable heteroplasmic sites in both tested tissues, only one instance of a changed PHP profile was observed in the buccal swab of the female individual MW-0080 (Dataset S14). By sequencing specimens from the initial sampling, *m.3586C>Y* was detected and subsequently confirmed in a replicate sequencing run with MAF 3.6%. In the second sampling after 10 months and the third sampling after an additional 14 months, there were no detectable levels of the minor allele, neither in MiSeq nor in NextSeq run at an average coverage of 1400X across all replicates. Although there is a systemic coverage drop flanking position 3586 in our sequencing workflow, the coverage is still sufficient for establishing loss of the T allele (SI Appendix, Results).

### PHP positions

To obtain the most comprehensive overview of all PHP positions and corresponding alternative alleles detected in this study, we aggregated results from all human subjects, from which technical (2 individuals) and biological (9 individuals) replicates were sequenced. The minor allele frequency for each PHP position identified in first-sampling specimens of MW-0002 and MW-0020 is given as an average value of technical replicates sequenced on MiSeq (Datasets S4-S7). For the remaining nine samples sequenced as biological replicates, MAFs obtained by sequencing first-sampling specimens are reported (Datasets S8-S16). In a total of 11 individuals, 62 heteroplasmic positions were identified: 28 were common to both tested tissues, 17 were found exclusively in buccal swabs and 17 were found exclusively in blood samples (Dataset S17).

Of all 62 identified positions, 29 are located in CR (25 in HV segments), 19 in coding region, nine in rRNA genes and five in tRNA genes. The distribution of PHP affected positions between the whole transcribed region (TR) (53%) and CR (47%) is as expected. It was shown that the PHP position count is higher in CR relative to its length, but the absolute PHP count is higher in TR [6, 8, 10], as summarized in [8]. The absolute PHP count in our dataset is minimally shifted towards TR, which is consistent with previous observations that the ratio of PHP positions in the transcribing region decreases in parallel with lowering the MAF detection threshold, summarized in [8]. The effect of the increased PHP abundance within CR at low MAF thresholds is further enhanced by the appearance of CR PHP positions with high relative substitution rates in more than one person (Table 1). In contrast, all TR PHPs are expectedly unique [6, 8, 10]. Therefore, in comparison to the PHP position count, the percentage of the total PHP count from both buccal and blood samples increases from 47 to 66% in CR. This indicates subtle mutational dynamics of the hypervariable region, present in at least some tissues, with low frequencies.

### Intra-individual PHP count

In the entire sample pool, which is biased towards the maximum PHP count per sample (SI Appendix, Methods), a total of 131 PHP events (single substitution in a single individual and tissue combination) were detected, of which 68 in buccal swabs and 63 in blood, averaging six PHPs per sample, for both tissues. All PHPs are either *C/T>Y* or *G/A>R* transitions, except for one sample with *m.5815A>M* transversion, found in both tissues with MAF > 12% (Dataset S17). The lack of transversions is consistent with previous findings in corresponding tissues [6, 10].

Sample MW-0087 had the highest cumulative PHP count (19 in both tissues) (Dataset S15). The highest tissue-specific PHP count was found in two blood samples (10 in each) and three buccal swab samples (9 in each) (Datasets S7, S9, S14 and S15). To evaluate this result in the context of previously published data summarized in [8], we adjusted MAF cut-offs comparable to the published studies as follows: 0.1% [6], 0.5% [10] and 10% [7]. Lowering thresholds for minor allele detection result in an increase in the number of detected PHPs at both the individual and population levels, although the exact correlation in various tissues is still largely unknown.

At the analysis threshold of 0.1%, the maximal intra-individual PHP count (10 in blood, 9 in buccal swabs) is similar to previously reported values: eight in blood and seven in buccal swabs found in two unrelated individuals [6]. Comparison of the positional PHP distributions with those reported in [6] reveals differences in reported bias between CR and TR. While Rebolledo-Jaramillo in [6] reports positional preference of PHPs towards TR, our findings establish strong PHP count bias in favor of CR in seven out of nine samples with ≥ 7 PHPs (Datasets S7, S9, S10, S12, S14 and S15). This is in agreement with the documented universal presence of low-level PHPs at highly polymorphic CR positions in healthy human subjects [4, 5]. Moreover, the affected loci in CR have high relative substitution rates and are, in some cases, tissue specific (Dataset 17). In the remaining two samples, PHPs are biased towards TR, with twofold (blood sample; Dataset S9) and six-fold (buccal swab; Dataset S10) excess. This might explain an unexpected relative lack of CR PHPs in [6], where only two samples with ≥ 7 PHPs were reported (one in blood and one in buccal swab), suggesting an insufficient sample size for reliable comparison.

As expected, the comparison at an MAF cut-off of 0.5% is more straightforward and exhibits a higher level of PHP count concordance. We restricted the comparison to blood, the only matching tissue reported across comparable datasets [6, 10]. The maximal intra-individual PHP count of four, observed in this study, five [6] and eight [10] is in accordance with the sample size (*N* = 11, 39, and 152, respectively). Finally, at an MAF cut-off of 10%, the maximal PHP count per individual was two (this study, [6, 10]) and three, as reported by Sanger-type sequencing of 588 subjects [7]. This indicates that the co-occurrence of three or more high-level PHPs (MAF > 10%) is a rare event.

While our dataset allows us to compare and confirm intra-individual PHP counts at various MAF thresholds, it is not suitable for assessing the general population-level incidence of high-PHP count individuals. Seven individuals were PHP positive at an MAF cut-off of 10%, and all 11 individuals were PHP positive at an MAF cut-off of 3%, which is a direct consequence of previously described biased sampling (SI Appendix, Methods).

### Inter-individual PHP count

The detected 131 PHP events (see above) range from completely unique (found in a single tissue of one individual) to those occurring across a combination of multiple individuals and/or tissues (Dataset S17). Testing the mesodermal (blood) and ectodermal (buccal swab) tissues allows the estimation of whether PHP mutations are of germ-line or somatic origin. We identified 33 pairs of PHPs in both blood and buccal swab of the same person: 35 PHPs exclusively in buccal swab, and 30 exclusively in blood. Of all 65 PHPs found exclusively in one tissue, only three had an MAF > 1%. This could indicate three possibilities: the single tissue low-level PHPs are somatic mutations that arose later in the life of an individual, with a smaller chance to expand (as compared to lifelong germline mutations); the corresponding PHPs were lost from one tissue during life; or the corresponding PHPs are present in the second tissue but at allele frequencies below the detection threshold of 0.1%. Indeed, in some instances, not all second tissue replicates had sufficient sequencing depth at a given position to reliably perform the call, therefore excluding the PHP from being reported. This scenario nevertheless implies a strong bias towards germline and/or early developmental mutations, which is in accordance with the predominant tissue co-occurrence at higher MAF values. On the other hand, given that single-tissue PHPs mostly occur at highly polymorphic positions in CR and exhibit tissue specificity across several individuals (Table 1), their occurrence is possibly explained as a somatic mutation maintained at a low-level in that tissue. Low-level PHP pedigree research is essential to clarify this and to evaluate implications of medical and forensic pertinence.

It is worth considering that “an echo” of a blood PHP in a buccal swab might originate from leukocytes in saliva, but our results do not corroborate this scenario; out of 33 tissue pairs, only six have a higher MAF in blood, and out of these, a single one with a blod:buccal swab MAF ratio sufficiently high (17-fold) to consider leukocyte contamination (Datasets S4 and S5). Even in this case, the buccal swab MAF remains stable over all replicates, which would not be likely in case of contamination.

Out of 62 reported PHP positions, 13 occur in more than one individual (Table 1). All of them reside within CR (five in HVS-I, six in HVS-II and two outside of hypervariable regions) and are predominantly highly polymorphic. This finding, along with the unique individual pattern of the TR PHPs (Dataset S17), is concordant with previous evidence of PHP hotspots in CR (e.g., pos. *m.152, m.189, m.204* and *m.16093*), and their almost universal absence in TR [6–8, 10].

### PHPs in mitochondrial genes

Within TR, we detected 44 PHP events at 33 positions, each found exclusively in a single individual, 11 of which were shared across both tissues (Dataset S17). Ten PHPs were found in *MT-RNR1* and *MT-RNR2* with MAF below 2% (seven in blood and three in buccal swabs). Just one individual had both tissues affected, which is also the only case where MAF exceeded 1%. In contrast to rRNA PHPs, seven out of eight tRNA PHPs are present in three individuals at high MAFs in both tissues (overall median value 16.5%). Interestingly, the single tRNA heteroplasmy *m.8363G>R* annotated as confirmed pathogenic [45] (accessed August 2023) is observed at low frequency in a single tissue. This finding is not unexpected, since the common presence of pathogenic mutations– kept at low levels by purifying selection in the general population– has been demonstrated and described previously [20, 47, 48]. In contrast to pathogenic alternative alleles, the observed expansion of, according to Mitomap [45], presumably benign alleles might reflect certain functional advantages of corresponding tRNAs.

We also identified 26 PHPs at 19 exclusive positions in protein coding genes: eight positions were affected by synonymous transitions, and 11 by non-synonymous transitions (five of them were predicted to be benign, while six were predicted to be probably damaging by PolyPhen-2 [49]) (Table 2). Similar counts of synonymous and non-synonymous PHPs have been documented previously [3, 7, 8]. The number of PHPs, their tissue distribution and MAF are concordant with the potential repercussions of allele substitution on protein function. First, synonymous and presumably benign non-synonymous PHPs together outnumber probably damaging non-synonymous PHPs. Second, in contrast to functionally neutral PHPs, damaging PHPs are found exclusively in either blood or buccal swabs, with comparably lower MAFs (and always < 1%) (Table 2). Even in such a small sample pool, the effect of purifying selection for possibly deleterious mutations is evident.

## Discussion

### *m.4929C>Y* in SRM-C

Low-level PHP found at position 4929 in SRM-C indicates almost complete expansion of the *m.4929C>T* variant in *MT-ND2*, which causes p.L154F amino acid residue replacement in the MT-ND2 protein. This replacement is predicted to be highly damaging by PolyPhen-2 [49], with scores of 0.999 according to HumDiv and 0.984 according to HumVar models [50]. Variant *m.4929C>T* is extremely rare in the human population– a single report found in Mitomap [45] (accessed August 2023) directs to ClinVar [51] entry (Accession VCV000692521.1) associated with Leigh syndrome, a severe neurological disorder with poor survival outcomes. Therefore, it is highly plausible that allele T in SRM-C is in fact a mutant variant, which almost entirely replaced the wild type C that remains detectable only as low-level PHP. It appears that the virus-immortalized lymphoblastoid cell culture line CHR, from which genomic material was extracted and commercialized under designation SRM® 2932 Component #1 CHR (SRM-C), displays resistance towards the deleterious effect of that particular amino acid change, which presumably is not the case in living human organisms. It would be interesting to test the functional effect of expanded, potentially deleterious variant of the gene whose product is an essential member of the electron transport chain and mutations of which are related to mitochondrial diseases [51]. Given all the evidence, the detected PHP call is likely genuine and probably induced through cell line derivation and/or culturing conditions.

Since all six PHPs detected in both SRM samples are rare or even previously unreported within human mitochondrial datasets, and are mostly found outside of the CR as non-synononymous alterations, it can be concluded that the PHP pattern in SRM controls deviates from the PHP pattern detected in human tissues. Therefore, further usage of SRM-H and SRM-C for research studies of human mitochondrial heteroplasmy should be carefully re-evaluated.

### Loss of *m.3586C>Y*

It is well documented that tissue-specific somatic mutations accumulate during a person’s lifetime, thus potentially influencing the profile of PHPs detected by MPS. By analysing various tissue types of many individuals with a broad age range, a positive correlation of PHP count to the person’s age is shown on a time scale of decades of human life [10]. However, longitudinal data on mitochondrial PHP in rapidly dividing cells, especially over shorter time periods, are scarce. Mutations in mtDNA not only accumulate with age but can also fluctuate in any direction and degree. The increased complexity of the mtDNA mutational landscape can be attributed to a combination of both random and directed processes not only within a tissue but also within a single cell, acting either by expanding or removing mutant molecules [2, 5, 10, 20, 52]. This in turn amplifies and diversifies the functional consequences of mtDNA mutations in terms of both severity and tissue localization. In any case, little is known about intra-individual temporal dynamics of mitochondrial heteroplasmy, which would be of great interest, especially for assessing potential forensic pertinence of PHP analysis.

In that context, loss of minor allele T at position 3586 (Dataset S14) is an important finding. Buccal swab samples contain predominantly squamous epithelial cells of ectodermal origin, with leukocytes of mesodermal origin mixed in varying proportions [53]. The detected minor allele T likely originates from epithelial cells since no corresponding reads were detected in any of the three sequenced blood samples from the same donor. For biological explanation, the dramatic eradication of *m.3586C>Y* PHP, within the 10-month interval between samplings, might be due to disadvantageous functional changes caused by non-synonymous mutation that alter the amino acid sequence (p.P94S) of MT-ND1. Although this particular amino acid change is predicted to be benign by PolyPhen-2 [49], there are no previous reports for the underlying nucleotide transition in the context of heteroplasmy and pathogenicity. Therefore, we can only speculate that the *m.3586C>Y* PHP has been subjected to negative selection in oral epithelium due to functional repercussions, considering limited knowledge on the selection of mtDNA single base substitutions, especially outside germline cells. The most extensive evidence on the substantial decrease in pathogenic mutations in mitotically active, differentiated tissues such as blood and epithelium was given by longitudinal tracking of the most common pathogenic mutation, *m.3243A>G*, in the *MT-TL1* gene [54–56]. It cannot be ruled out that the *m.3586C>T* mutation follows a similar pattern. Regardless of the underlying mechanism, this finding could become forensically relevant for future applications because it indicates the possibility of altered PHP signature between repeated, time-lapsed sampling of mitotically active tissues such as epithelium, blood or sperm of the same individual.

### Position *m.16093*

Regardless of its relatively low substitution rate, CR heteroplasmy *m.16093T>Y* has often been detected as the most represented PHP across numerous datasets [8]. As such, it became a *de facto* benchmark in assessing the authenticity of PHP results within a particular dataset. However, in datasets produced below 1% MAF cut-offs, the most abundant PHP shifts towards other CR positions [5, 6, 10]. In our dataset, produced at a comparably low MAF cut-off, we identified as many as six CR positions, with PHPs detected in more individuals than *m.16093T>Y* (Table 1). Median MAFs at these positions do not exceed 0.5%, whereas a total of six PHPs at *m.16093* observed in both tissues of three individuals have a median MAF of 3%. This is the highest median MAF value in a wider context of all PHPs detected more than once at a single position in the entire dataset (Table 1). The pattern of occurring PHP positions indicates that position *m.16093* is indeed susceptible to mutational events. However, when observed in the context of MAF and positional substitution rates, the prevalence of *m.16093* is better explained by its higher propensity for the expansion of an alternative allele, rather than mutational rate.

The evidence from multiple tissue studies suggests that *m.16093* PHP is often present in all tested tissues of an individual, with high MAFs [5, 10, 15, 57]. This also contributes to the finding of the highest total *m.16093* PHP count within a particular dataset, unless a low MAF cut-off is applied. Although we tested only two tissues, the described feature is evident in our dataset as well; with the increase of MAF cut-off from 0.1 to 1%, *m.16093T>Y* becomes the most abundant PHP, at both inter-individual level (three persons affected) and overall (total count of six) (Tables 1 and Dataset S17).

It is noteworthy that four PHP positions (*m.146*, *m.152*, *m.16129* and *m.16519*), found in more individuals than *m.16093* at MAF 0.1%, are at the same time more polymorphic than *m.16093* (Table 1). This implies that the apparent discrepancy between the relatively low substitution rate and the highest PHP abundance of position 16093 across datasets becomes less prominent at lower MAF cut-offs. This observation is yet another argument for reconsidering *m.16093* PHP count as a dataset quality benchmark, especially for high coverage MPS studies.

### Low-level PHPs

Positions affected by low-level substitutions are distributed across the entire mtDNA, but hotspots are limited to highly polymorphic positions in the CR (Table 1). Clustering of detected PHPs at hotspots, their reproducibility across replicates and previous evidence of universal presence in healthy humans [4] argue in favour of their plausibility. In addition, it is unlikely that the sequencing error patterns would completely coincide with known mutational hotspots within CR.

The remaining alternative hypothesis for misinterpretation of low-level PHP would be coamplification of NUMT regions, which is considerably prevented by library preparation strategy [34] (SI Appendix, Methods). Postexperimental confirmation of the absence of NUMT coamplification can be inferred from the lack of previously unreported minor alleles within the dataset because 74% of alternative NUMT alleles are originally not present among modern human mtDNAs [58]. Of 62 identified PHP positions, minor alleles at only five of them are not reported as contemporary variants in Mitomap [45] (accessed August 2023; *m.1486T*, *m.2032A*, *m.2673A*, *m.9622T*, *m.14898C*). It is quite possible that these protein coding and rRNA gene variants could not expand to fixation. For example, *m.9622C>T* and *m.14898T>C* are non-synonymous transitions predicted to be probably damaging by PolyPhen-2 [49] (Table 2). Additionally, of these five minor alleles, only *m.2673G>A* and *m.9622C>T* are known NUMT variants according to [58]. However, NUMT fragments containing variants in question are < 1 kb in length, which is well below the coamplifying capacity of primers used in this study.

We detected a number of low-level PHPs that exhibit tissue specificity. This is yet another indicator of NUMT-uncompromised PHP calls because highly reproducible NUMT coamplification from nDNA in one tissue would be expected in another, repeatedly analysed tissue of the same individual. The tissue specificity of PHPs was documented previously, predominantly in more medically than forensically relevant tissues, such as muscle, liver and brain, at MAF > 1% [10, 15]. In contrast, we found tissue specificity only at MAF < 1%: *m.146T>Y* exclusively in blood samples of four individuals; *m.16129G>R* exclusively in blood of six individuals; and *m.16293A>R* exclusively in buccal swabs of five individuals (Dataset S17). In addition, we found *m.152T>Y* and *m.189A>R* more often in buccal swabs than in blood (five vs. two, for both positions). Confirmation of low-level tissue-specific PHPs was sought in the most comparable study of matching tissues at an MAF of 0.1% [6]. Although direct confirmation was not found, it is noteworthy that the aim of [6] was to investigate maternal PHP inheritance, and PHPs with MAF < 1% were only reported if the other tissue sample in mother-child duo had corresponding PHP above MAF 1%. This explains the absence of PHPs at positions *m.146*, *m.16129* and *m.16293*, which we detected exclusively in one tissue and always with an MAF < 1%.

We also observed co-occurrence of two low-level PHP pairs: *m.146T>Y/m.16129G>R* in the blood of three individuals and *m.152T>Y/m.16293A>R* in the buccal swabs of four individuals (Dataset S17). The phenomenon of PHP linkage was first described in [10], where nine PHP position pairs were identified more often than expected by chance in various tissues. Similarly, these positions are almost exclusively found in CR (eight out of nine pairs), which would indicate some kind of involvement in mtDNA replication and might reflect tissue-specific requirements for mtDNA propagation. It is also worth noting that presumably linked positions correspond to the location of 7S DNA, which is the third strand “player” in the D-loop, and thus possibly contributes to minor allele-containing reads. This finding might hint towards the existence of a “PHP microhaplotype” in blood and buccal swabs, which could potentially be used as a tissue-type marker.

## Conclusions

In this study, we performed high-coverage sequencing of whole mitochondrial genomes in longitudinal blood and buccal swab samples from 11 human donors. We found a high abundance of low-level PHPs (MAF < 1%) in the mitochondrial DNA control region (CR), their distinct tissue-specific patterns and minor allele linkage. We also explored the position-dependent potential of minor allele expansion in PHPs, and short-term PHP instability in a mitotically active tissue of oral epithelium.

Diverse expansion potential of point mutations at different positions of mitochondrial genome leads to unique PHP distribution patterns at low minor allele frequency thresholds. PHPs with MAF below 1% are common, mostly affect highly polymorphic positions in the control region, and exhibit linkage and tissue specificity, as well as instability in mitotically active tissues. PHP patterns in continuously cultured human cells do not resemble those found in vivo, which hinders a direct comparison of the two systems. Distinct patterns of the low-level PHPs observed across tissues and individuals, provide evidence for future use in medicine as molecular biomarkers, or forensics in questions of identity, tissue-specificity, age and maternal kinship.

### Electronic supplementary material

Below is the link to the electronic supplementary material.

Supplementary Material 1

Supplementary Material 2

## Data Availability

The datasets generated during and/or analysed during the current study are available from the corresponding author upon reasonable request.
