# Supplementary material for: Mitochondrial point heteroplasmy: insights from deep-sequencing of human replicate samples
Source: BMC Genomics. 2024 Jan 10;25:48. doi: 10.1186/s12864-024-09963-z (PMC10782721; doi:10.1186/s12864-024-09963-z)
Supplement: Supplementary file 1 — Supplementary Material 1 [file 12864_2024_9963_MOESM1_ESM.docx]

Supplementary Information (SI)

**Mitochondrial point heteroplasmy: insights from deep-sequencing of replicate samples**

Marina Korolija, Viktorija Sukser, Kristian Vlahoviček

Correspondence: Marina Korolija

Email: [mkorolija@mup.hr](mailto:mkorolija@mup.hr)

This PDF file includes:

Supporting text

Legends for Datasets S1 to S17

SI References

Other supporting materials for this manuscript include the following:

Datasets S1 to S17

Supporting Information Text

Methods

Library preparation, sequencing and data analysis

Upon obtaining informed consent, blood and buccal swab samples were collected, and total DNA was extracted from 11 donors, and the Standard Reference Material® (SRM®) 2392 Component #1 CHR (abbreviated as SRM-C) [1] and SRM® 2392-I HL-60 (abbreviated as SRM-H) [2], as described [3].

Target enrichment, library preparation, sequencing and data analysis were performed according to the Illumina Human mtDNA Genome Guide. (https://emea.support.illumina.com/downloads/human_mtdna_genome_guide_15037958.html) with modifications described previously [3, 4]. Briefly, mitochondrial genome was amplified in two overlapping PCR fragments of 9.1 kbp and 11.2 kbp, with the overlap covering the entire mtDNA control region (CR; approx. 1.1 kbp). The long-range PCR mtDNA enrichment approach was chosen for it is known as the most effective in avoiding NUMT co-amplification [5]. Size and specificity of PCR products were visually assessed on agarose gel. Upon quantification, normalization and pooling corresponding mtDNA amplicons, libraries were prepared using Nextera® XT Library Prep Kit (Illumina, San Diego, CA, USA). Normalized libraries were pooled in batches of 24-48 samples per run, with a 5% spike-in of PhiX Sequencing Control v3 (Illumina, San Diego, CA, USA). Paired-end sequencing was performed on Illumina® MiSeq FGxTM instrument using MiSeq® Reagent Kit v2, standard flow-cell, 300 cycles (2 x 151 bp).

Technical replicate libraries were prepared using Nextera® XT Library Prep Kit from the same PCR amplicons, and these were used to validate our MiSeqFGxTM workflow by an independent laboratory on Illumina® NextSeq®500 platform. NextSeq®500/550 Mid Output Kit v2.5, 150 cycles, was used for paired-end sequencing (2 x 75 bp). Resulting point heteroplasmies (PHPs) from both MPS platforms were compared to each other for concordance [3].

Data analysis was performed by uploading fastq files generated by MiSeq® Reporter v.2.5.1.3 (Illumina®) to Illumina® BaseSpace® Sequence Hub online platform, where they were processed by BaseSpace® mtDNA Variant Processor v1.0.0 App. Common settings of minimum basecall quality score for a call = 30 and genome used for alignment = rCRS (revised Cambridge reference sequence) [6, 7] were applied to all analyses. At all stages of analysis, samples were visually inspected in BaseSpace® mtDNA Variant Analyzer v1.0.0 App, which allowed review of coverage profiles and sequences, as well as export of Excel-format reports used for final variant confirmation and comparison of samples [3].

Data analysis was performed aslo by mapping raw fastq reads to the reference rCRS genome [6, 7] using the bwa program version 0.7.12-r1039 [8], with the ‘mem’ algorithm and default parameters. Samtools v1.8 [9] was used for converting the mapped reads to BAM format, fixing mate-pair information, sorting and indexing the resulting files. Indels were left-aligned with the GATK v4.0.3.0 [10] LeftAlignIndels command against the rCRS reference fasta file, and subsequently the PCR duplicates were removed with Samtools. Reads were filtered using Bcftools v1.8 [11], with minimum base quality of 20 and mapping quality of 30. Base frequencies at each reference position were counted into a VCF file, normalized by left-aligning indels, and multiallelic variants split into separate rows, producing final data format for manual review. All reported PHP positions were manually reviewed by inspecting respective BAM files in Integrative Genomics Viewer (IGV) tool v.2.4.16 [12, 13].

Additional criteria for PHP conformation

Not strictly exclusive, but rather indicative evaluation criteria were applied to assess each reported PHP: strand balance, sequence environment, previously reported variants at the same position - crosschecked via Mitomat database [14] - presence of a particular PHP in other tested tissue type, transition to transversion ratio and synonymous versus nonsynonymous mutation count. The issue of strand balance for PHP positions was addressed by calculating strand bias (SB) score for minor alleles in all sequenced samples, according to the formula previously used in [15]. PHP sites with SB score > 1 in all or majority of replicates were filtered out, while SB score > 1 when find in minority of replicates of reported PHP sites are highlighted in red (Datasets S3-S16). Sequence environment flanking each PHP was individually inspected for error-prone motifs (homopolymer nucleotide stretches, dinucleotide or other repeat motifs, and other low-complexity regions). To reduce the risk of false positives, all reported PHP positions were manually reviewed by inspecting respective BAM files in Integrative Genomics Viewer (IGV) tool v.2.4.16 [13, 16]. To assess the frequency of potentially pathogenic, non-synonymous minor alleles at reported PHP positions, PolyPhen-2 tool was used [17]. The predictive impact of amino acid substitution on the structure and function of human protein was described with probability scores (HumDiv and HumVar) ranging from 0 to 1 (benign to damaging, respectively) [18].

To further confirm plausibility of the reported PHPs, phylogenetic criterion of nucleotide substitution rate was applied. It was shown previously that PHP frequency in a population corresponds with substitution rate for mtDNA positions [19–22]. It is therefore expected that PHP incidence at high substitution rate positions is also high. In Table S3 of [23], substitution rate for each human mtDNA position is described by whole number value ranging from 0 (for the least, or presumably non-polymorphic positions) to 209 (for the most polymorphic, position 16519). Numerical values correspond to the number of occurrences of a particular variant at certain mtDNA positions found within dataset of 2196 complete human and 32 primate mitochondrial genomes [23]. Of 16,569 positions in total, approximately 3,800 have at least one substitution detected in the described dataset [23].

Reported PHPs were also compared to the previous, most comprehensive MPS PHP study of 12 tissues from 152 individuals [19], which is indicated in **Table 1** and **Datasets S2-S7**. PHPs detected in more than one person were additionally cross-checked with PHP report from [24] (**Table 1**).

Finally, to avoid NUMT co-amplification and subsequent identification of NUMT-derived, instead of mtDNA-derived variants as low-level minor alleles, long-range PCR enrichment strategy was opted for. Since only 0.8% of all known NUMTs are >10 kb in length [25, 26], long mtDNA amplicon generation is the most effective approach in avoiding NUMT co-amplification [5] . It has been shown that mtDNA enrichment primers that produce shorter amplicons readily co-amplify NUMTs, whose reads contain high number of phased variants [26]. Therefore, it was assured by visually inspecting individual reads in IGV, that reported minor alleles do not originate from those containing multiple (phased) variants. Additionally, the mtDNA stretch from nucleotide positions 650-850 was examined, because this particular region is prone to NUMT interference [25–27]. No evidence of minor variants within that region in any of analysed samples was found.

Patterns of erroneous low-level PHP calls in MiSeqFGx and NextSeq results

In the course of heteroplasmy-specific analyses performed at lower analysis thresholds in BaseSpace mtDNA applications (i.e. AT = 0.1%, IT = 0.1%, and minimum read count = 10), we observed patterns of low-level PHP signals that arose with high occurrence in sequencing results of both MiSeqFGx and NextSeq platforms. Of particular interest were positions where PHP calls were detected across different sequencing runs, different sample types and samples originating from different persons. Such positions, appearing in >80% of all MiSeqFGx and >63% of all NextSeq validation samples, are potential indicators of system errors.

MiSeqFGx results displayed the following pattern: *m.2487A>M*, *m.3447A>M*, *m.3492A>M*, *m.8512A>M*, and *8577A>M*. In all instances, detected heteroplasmy call was M (mixture of bases A and C), with A being the major allele, and percentage of minor allele C ranging from 0.1-7.2%. However, it is unlikely these would be mistaken for true PHP calls due to their low GQ values (<37), and also because all these positions include adenine that precedes a stretch of three or more cytosines. Interestingly, A-C transversions have been previously reported as the most common Illumina sequencing errors [28].

In NextSeq results the following pattern was observed: range 538-574 (multiple positions with call M PHPs), *m.1900A>M*, *m.6538A>M*, *m.6565M*, *m.6583A>M*, *m.8129A>M*, *m.8138A>M*, *m.8144A>M*, *m.12391A>M*, *m.12400A>M*, *m.14280A>M*, *m.15266A>M*, *m.15536A>M*, and *m.16399A>M*. Again, in all cases A is the major allele, while C represents minor allele ranging from 0.1-2.4%; sequence environment is highly similar to the one described above for MiSeq results: PHP occurring at a position where A either precedes or intercalates into C stretches. Like before, these positions would unlikely be called as true PHPs, since their GQ values are low (<37).

Besides these patterns identified across all validation samples, distinctive for MiSeq and for NextSeq results, there were four other presumable PHPs (*m.2623A>R*, *m.3244G>R*, *m.10320G>K* and *m.15799A>W*), appearing sometimes serially and sometimes completely randomly across different tissue types, samples of different persons, and different sequencing runs. These four positions are present in both MiSeq and NextSeq results, with 38-57% and 46-83% samples affected, respectively. In contrast to previously described erroneous PHP positions, these four exhibit high GQ values (>45) and seemingly non-problematic sequence environment, which makes them more difficult to exclude from the PHP report. However, it is extremely unlikely that the described pattern is of biological origin, because of virtually non-existing substitution rates for mentioned positions (only one occurrence for *m.3244G>R*) [23] and the absence of any PHP event >1% within the dataset of >300 individuals sequenced in our laboratory. Another strong argument against biological authenticity of transversions (*m.10320G>K* and *m.15799A>W*) detected in multiple samples, is pronounced transition bias that is generally present in human mitochondrial DNA. Therefore, *m.2623A>R*, *m.3244G>R*, *m.10320G>K* and *m.15799A>W* should also be included as a part of error patterns in both MiSeq and NextSeq sequencing results. It is interesting to note that the incidence of these four PHPs in SRMs, deviates from other validation samples, on both sequencing platforms: SRM-H contains none of the four PHPs, while in SRM-C only *m.15799A>W* is found. So, it is perhaps not unfounded to speculate that, at least regarding these four PHPs, origin of SRMs from cell culture lines could be the source of this biased pattern, as opposed to fresh tissue samples collected from donors.

Indel calls were largely disregarded since they require additional, specific bioinformatic processing. Despite that, we noted high occurrence of indels (64-100% of all validation samples) in both MiSeqFGx and NextSeq results for the following positions: *m.71, m.5752, m.6698, m.11038, m.12245 and m.13237*. Low percentage of deletion or insertion calls in these mtDNA coordinates were detected in homopolymer stretches of A or G (most commonly 6-8 identical bases). However, these calls are most likely products of alignment issues, i.e. bioinformatic artefact, rather than true biological state within the samples, particularly since some indels are omnipresent, which is not biologically viable.

In general, distinct patterns of false positive low-level PHP calls were identified for MiSeqFGx and for NextSeq sequencing results. While positions differ, the signal detected is predominantly a mixture of major allele A and minor allele C, occurring where A precedes or intercalates relatively long stretches of cytosines. This suggests that Illumina chemistry, or possibly detection method on instrument, might struggle with such regions. It is important to identify these problematic regions and patterns, in order to eliminate them as false positive signals and ensure reliable PHP calls.

Heteroplasmies *m.2623A>R* and *m.3244G>R*, which were detected reproducibly in three individuals, were discarded despite fulfilling the previously described filtering criteria. These PHPs were recognized as artefacts because they serially occurred within the dataset, across the majority of replicates, and so defy biological sense and previous observations. PHPs at these two positions were the only recognizable artefacts that could not be filtered out by our approach; this indicates that some erroneous variant calls can still be detected reproducibly and with high enough Q scores, just like true minor alleles. However, both positions are surrounded by error-prone immediate sequence environment, so caution and thorough inspection of all low-level PHPs occurring there is necessary.

Sequencing of biological replicates (longitudinal study)

Sequencing of blood and buccal swab samples taken in time intervals as biological replicates was performed, from selected nine individuals who exhibited one to three PHPs above previously calculated INT-AT threshold of 3% [3]**.** Selection was intentionally biased towards samples with the highest PHP count, in order to provide as many observation points as possible for temporal follow-up. Detected PHP profiles are therefore not representative for the general population.

Each of nine individuals (MW-00: 12, 26, 65, 67, 73, 78, 80, 87 and 88), were sampled three times, with 10-month and 14-month intervals between samplings for buccal swabs, and with 5-month and 14-month intervals for blood specimens (**Dataset S1**). Samples of both tissues obtained in the first sampling underwent mtDNA enrichment in two independent long-range PCR amplifications, and these technical duplicates were sequenced in separate runs. Samples from the second sampling were sequenced once, whereas samples from the third sampling underwent library preparation from the same PCR amplicons in duplicate, and were sequenced in separate runs (on MiSeqFGx and on NextSeq). The aim of this strategy was to avoid PCR-introduced, run-specific and platform-specific errors. PHPs detected in both replicates from the first sampling were monitored in subsequent, tissue-matching samples of the same individual. Additionally, potential gain of PHP in the second and/or third sampling was also monitored. Since the gain or loss of PHP below noise threshold is even more challenging than low-level PHP authentication, only PHPs with minor allele frequency (MAF) ≥3%, detected in at least one biological replicate, were monitored.

For reporting all PHPs found in nine individuals, including PHPs with MAF 0.1-3% (**Dataset S17**), the condition of presence in all five technical and biological replicates per sample after data filtering as described, was set. Since all detected PHPs with MAF ≥1% were always present in all five replicates of the sample, the term “low-level” in this study refers to PHPs with MAF <1%.

Results

Replicates from MW-0002

In buccal swab of female donor MW-0002, two completely reproducible low-level PHPs were found: *m.16298T>Y (*average MAF 0.3%) and *m.16311T>Y* (average MAF 0.4%) **(Dataset S4)**. Both positions reside within mtDNA hypervariable region 1 (HVS-I), and are highly polymorphic [23]. Variant *m.16298T>Y* was previously reported in several of the 12 examined tissues from six persons above the age of 45 years, with the majority of tissues ranging in MAF from 0.5% to 1% [19]. Only intestine and skeletal muscle exhibited MAFs of 1.0-12.5%, indicating positive selection of that particular PHP in these tissues [19]. MW-0002 donor is considerably younger (27 years old), and thus unlikely *m.16298T>Y* positive. However, MAF detection threshold in previous study was 0.5%, which prevented detection of PHPs in younger individuals with presumably lower mutational load. Our finding of *m.16298T>Y* with an average MAF of 0.3%, corroborates that scenario. It is on the other hand unexpected, that from all investigated tissues [19], there is just one *m.16298T>Y* found in skin, whose epithelial component is ontogenically closest to oral epithelium of buccal swab. Perhaps deeper skin layers originating from mesoderm are simply the primary contributors to PHP pattern in highly heterogeneous skin sample.

In the context of the dataset in this study, low-level heteroplasmy *m.16298T>Y* is found solely in individual MW-0002, which might exclude the possibility of systematically occurring error. However, exclusion is somewhat difficult since, out of 11 sequenced individuals, only MW-0002 has C instead of T as major variant at position m.16298. Confirmation for this PHP was found within our internal population database, where *m.16298T>Y* is found in several buccal swabs with the highest MAF of 1.1%.

Heteroplasmy *m.16311T>Y* is another low-level event, reproducibly detected in buccal swab of MW-0002, with its validity corroborated in blood. Apart from very high variability of the position and local nucleotide environment that is not known to cause sequencing artefacts, reported PHP is found in blood of the same individual with average MAF of 6.7% **(Dataset S5)**, which is well above sequencing noise range. Minor variant *m.16311T>Y* is found in two more individuals in this study, with MAFs averaging at 0.3% (C in blood of MW-0020 **(Dataset S7)** and T in buccal swab of MW-0026 **(Dataset S9)**, which might seem indicative of a system artefact. However, given the high substitution rate for the position m.16311, and the opposite direction of mutations found in two additional samples with the same PHP call, it is possible that few copies of mtDNA molecule with point mutations at HV sites are commonly present within multiple or specific tissues [29, 30]. These occurrences regularly remain undetected with commonly used heteroplasmy detection thresholds, so this possibility remains to be re-confirmed in the future.

In blood samples of MW-0002, the second PHP, *m.16129G>R*, was found with average MAF of 0.2% (**Dataset S5**). Again, the position is extremely polymorphic, residing within presumably error-proof nucleotide environment in HVS-I, and reported previously as heteroplasmic with both A and G as minor alleles [9, 14]. Another parallel with *m.16311T>Y* is high inter-individual prevalence, which is even more pronounced for *m.16129G>R*, with as much as seven positive individuals out of 11 tested. In contrast to *m.16311T>Y*, m.16129G>R exhibits blood-specificity, with just one finding of low-level m.16129 PHP in buccal swab, but with minor allele G (**Datasets S9, S17**). This hints at tissue-specific direction of PHP mutation, which is yet to be investigated. Another interesting observation discussed in the main text is co-occurrence of *m.16311T>Y* with low-level *m.146T>Y* in blood.

Replicates from MW-0020

In buccal swab of female donor MW-0020, which was sequenced most extensively and used as an internal control sample, we reproducibly detected four PHPs, with frequencies of minor alleles ≤7% (**Dataset S6**). In addition to PHPs found at highly polymorphic positions residing within HVS-I (*m.16293A>R*) and HVS-II (*m.152T>Y*), we identified two positions within *MT-CO1* and *MT-CO3* affected by non-synonymous transitions, predicted as benign by PolyPhen-2 [17, 18]. Both coding region PHPs (*m.7348T>Y*, and *m.9325T>Y*) are present in blood of the same individual at comparable frequencies (**Dataset S7**), indicating germline or early developmental mutations. In both cases, alternative allele was previously annotated as single allele variant, but there are no matching reports of heteroplasmy [14, 19]. It is well established that the transcribing region (TR) mutations at population level do not cluster at particular positions as they do in CR, but are rather scattered across the mitogenome, and are often unique within large datasets [19, 31]. Therefore, the lack of previous reports for PHPs *m.7348T>Y* and *m.9325T>Y* does not diminish the high probability of their authenticity, given that they are completely reproducible across many replicates, that they reside within straightforward sequence environment, possess relatively high MAF (especially for position *m.9325*), and co-occur in both tissue types.

In contrast to TR PHPs and *m.152T>Y* in HVS-II, another CR PHP in this sample – *m.16293A>R* (with average MAF of 0.2%) is much more challenging to qualify as authentic (**Dataset S6**). Although the position exhibits high relative substitution rate [23], and the local sequence does not imply erroneous detection of minor allele, there are no heteroplasmy reports for that position in [19], except for one individual, where minor alleles are present within six out of seven PHP positive tissues with frequencies below 1.2%. Considering that m.16293A>R was found with MAF mostly ≤0.5% in as much as five out of eleven tested individuals (**Dataset S17**), there is a possibility that this particular PHP is common, with an unknown mechanism of maintaining G variant at low level. That would also explain absence of previous PHP reports for otherwise highly polymorphic position.

Apart from three shared PHPs found in both buccal swab and blood, seven more are detected exclusively in blood of the donor MW-0020 (**Dataset S7**). All blood PHPs, except *m.9325T>Y*, exhibit MAF <1%, again indicating non-permissive conditions for mutation to reach readily detectable levels. This particular blood sample, along with blood sample of the donor MW-0087 **(Dataset S15)** exhibits the highest PHP count per sample (ten), which is consistent with maximal intra-individual counts found at similar MAF thresholds [19, 24]. It is noteworthy that MW-0020 blood sample contains low-level tRNA heteroplasmy (*m.8363G>R*, MAF 0.4%), which is annotated as confirmed pathogenic [14] (accessed August 2023). This is in accordance with previously demonstrated common presence of pathogenic mutations, kept at low levels by purifying selection, at population level [32–34].

Longitudinal PHP study – *m.3586* sequencing coverage drop

Since the frequency of minor allele in the first-sampling specimen is above sequencing noise threshold, validity of *m.3586C>Y* is solid. However, there is an evident coverage drop at position m.3586, with the lowest read depth values among all PHP positions identified and reported in this study. Sequence context of position m.3586 exhibits low complexity, with several consecutive C tracts, which presumably manifests as the lack of properly aligned reads resulting in coverage drop. It is worth noting that the described drop roughly spans positions m.3400-3600, and is evident in all sequenced samples, regardless of run or tissue type [3]. This is also the case for several other mtDNA regions, some of which are of low sequence complexity as well. Nonetheless, immediate flanking region of position 3586 does not indicate erroneous detection of T as an alternative allele, particularly not at such high frequencies. We therefore consider average coverage of 1400X across MW-0080 replicates sufficient for reliable detection of the alternative allele, and so PHP identified in the first-sampling specimen (*m.3586C>Y*) is designated as authentic.

Absence of m.3586C>T variant across databases might favor assumption of its biological adversity. There are only two sequences with *m.3586C>T* variant, one of the phylogenetically distant haplogroup C (GenBank accession KY680984.1) and the other from ancient DNA (GenBank accession MH043578.1) annotated in Mitomap [14] (accessed August 2023). Both of these sequences, in contrast to the haplotype of our MW-0080 sample, contain additional non-synonymous variants that might correct potential deleterious effect of *m.3586C>T* on protein function.

Legends for Datasets (separate file)

Dataset S1. Human participants and summary of sequencing experiments covered by this study. Sequencing runs performed on Illumina® instruments MiSeq FGxTM and NextSeq500®.

Dataset S2. Reproducible point heteroplasmies (PHP) in all replicates of NIST Standard Reference Material® 2392-I HL-60. Results are shown for both BaseSpace mtDNA analysis applications and our in-house bioinformatic mtDNA analysis pipeline.

Dataset S3. Reproducible point heteroplasmies (PHP) in all replicates of NIST Standard Reference Material® 2392 CHR. Results are shown for both BaseSpace mtDNA analysis applications and our in-house bioinformatic mtDNA analysis pipeline.

Dataset S4. Reproducible point heteroplasmies (PHP) in all replicates of buccal swab samples from individual MW-0002. Results are shown for both BaseSpace mtDNA analysis applications and our in-house bioinformatic mtDNA analysis pipeline.

Dataset S5. Reproducible point heteroplasmies (PHP) in all replicates of blood samples from individual MW-0002. Results are shown for both BaseSpace mtDNA analysis applications and our in-house bioinformatic mtDNA analysis pipeline.

Dataset S6. Reproducible point heteroplasmies (PHP) in all replicates of buccal swab samples from individual MW-0020. Results are shown for both BaseSpace mtDNA analysis applications and our in-house bioinformatic mtDNA analysis pipeline.

Dataset S7. Reproducible point heteroplasmies (PHP) in all replicates of blood samples from individual MW-0020. Results are shown for both BaseSpace mtDNA analysis applications and our in-house bioinformatic mtDNA analysis pipeline.

Dataset S8. Reproducible point heteroplasmies (PHP) in samples from individual MW-0012. Both sample types (buccal swabs and blood on FTA cards) were sampled three times from this individual. Results are shown for both BaseSpace mtDNA analysis applications and our in-house bioinformatic mtDNA analysis pipeline.

Dataset S9. Reproducible point heteroplasmies (PHP) in samples from individual MW-0026. Both sample types (buccal swabs and blood on FTA cards) were sampled three times from this individual. Results are shown for both BaseSpace mtDNA analysis applications and our in-house bioinformatic mtDNA analysis pipeline.

Dataset S10. Reproducible point heteroplasmies (PHP) in samples from individual MW-0065. Both sample types (buccal swabs and blood on FTA cards) were sampled three times from this individual. Results are shown for both BaseSpace mtDNA analysis applications and our in-house bioinformatic mtDNA analysis pipeline.

Dataset S11. Reproducible point heteroplasmies (PHP) in samples from individual MW-0067. Both sample types (buccal swabs and blood on FTA cards) were sampled three times from this individual. Results are shown for both BaseSpace mtDNA analysis applications and our in-house bioinformatic mtDNA analysis pipeline.

Dataset S12. Reproducible point heteroplasmies (PHP) in samples from individual MW-0073. Both sample types (buccal swabs and blood on FTA cards) were sampled three times from this individual. Results are shown for both BaseSpace mtDNA analysis applications and our in-house bioinformatic mtDNA analysis pipeline.

Dataset S13. Reproducible point heteroplasmies (PHP) in samples from individual MW-0078. Both sample types (buccal swabs and blood on FTA cards) were sampled three times from this individual. Results are shown for both BaseSpace mtDNA analysis applications and our in-house bioinformatic mtDNA analysis pipeline.

Dataset S14. Reproducible point heteroplasmies (PHP) in samples from individual MW-0080. Both sample types (buccal swabs and blood on FTA cards) were sampled three times from this individual. Results are shown for both BaseSpace mtDNA analysis applications and our in-house bioinformatic mtDNA analysis pipeline.

Dataset S15. Reproducible point heteroplasmies (PHP) in samples from individual MW-0087. Both sample types (buccal swabs and blood on FTA cards) were sampled three times from this individual. Results are shown for both BaseSpace mtDNA analysis applications and our in-house bioinformatic mtDNA analysis pipeline.

Dataset S16. Reproducible point heteroplasmies (PHP) in samples from individual MW-0088. Both sample types (buccal swabs and blood on FTA cards) were sampled three times from this individual. Results are shown for both BaseSpace mtDNA analysis applications and our in-house bioinformatic mtDNA analysis pipeline.

Dataset S17. List of all point heteroplasmies detected in buccal epithelium and blood samples of 11 individuals when analyzed at low thresholds (i.e. analysis and interpretation thresholds = 0.1%).

SI ****References****

1. Tarlov MJ, Choquette SJ. Standard Reference Material 2392, Certificate of Analysis. 2018.

2. Viliker MJ, Rumble JJr. Standard Reference Material 2392-I, Certificate of Analysis. 2003.

3. Sukser V, Rokić F, Barbarić L, Korolija M. Assessment of Illumina® Human mtDNA Genome assay: workflow evaluation with development of analysis and interpretation guidelines. Int J Legal Med. 2021;135:1161–78.

4. Sukser V, Korolija M, Račić I, Rožić S, Barbarić L. Human whole mitochondrial genome sequencing and analysis: optimization of the experimental workflow. Croat Med J. 2022;63:224–30.

5. Li M, Stoneking M. A new approach for detecting low-level mutations in next-generation sequence data. Genome Biol. 2012;13:R34.

6. Anderson S, Bankier AT, Barrell BG, de Bruijn MH, Coulson AR, Drouin J, et al. Sequence and organization of the human mitochondrial genome. Nature. 1981;290:457–65.

7. Andrews RM, Kubacka I, Chinnery PF, Lightowlers RN, Turnbull DM, Howell N. Reanalysis and revision of the Cambridge reference sequence for human mitochondrial DNA. Nat Genet. 1999;23:147.

8. Li H, Durbin R. Fast and accurate short read alignment with Burrows-Wheeler transform. Bioinformatics. 2009;25:1754–60.

9. Li H, Handsaker B, Wysoker A, Fennell T, Ruan J, Homer N, et al. The Sequence Alignment/Map format and SAMtools. Bioinformatics. 2009;25:2078–9.

10. McKenna A, Hanna M, Banks E, Sivachenko A, Cibulskis K, Kernytsky A, et al. The Genome Analysis Toolkit: a MapReduce framework for analyzing next-generation DNA sequencing data. Genome Res. 2010;20:1297–303.

11. Danecek P, Auton A, Abecasis G, Albers CA, Banks E, DePristo MA, et al. The variant call format and VCFtools. Bioinformatics. 2011;27:2156–8.

12. Robinson JT, Thorvaldsdottir H, Winckler W, Guttman M, Lander ES, Getz G, et al. Integrative genomics viewer. Nat Biotechnol. 2011;29:24–6.

13. Robinson JT, Thorvaldsdóttir H, Wenger AM, Zehir A, Mesirov JP. Variant Review with the Integrative Genomics Viewer. Cancer Research. 2017;77:e31–4.

14. Lott MT, Leipzig JN, Derbeneva O, Xie HM, Chalkia D, Sarmady M, et al. mtDNA Variation and Analysis Using Mitomap and Mitomaster. Curr Protoc Bioinformatics. 2013;44:1 23 1-26.

15. Guo Y, Li J, Li CI, Long J, Samuels DC, Shyr Y. The effect of strand bias in Illumina short-read sequencing data. BMC Genomics. 2012;13:666.

16. Robinson JT, Thorvaldsdóttir H, Winckler W, Guttman M, Lander ES, Getz G, et al. Integrative genomics viewer. Nat Biotechnol. 2011;29:24–6.

17. Adzhubei IA, Schmidt S, Peshkin L, Ramensky VE, Gerasimova A, Bork P, et al. A method and server for predicting damaging missense mutations. Nat Methods. 2010;7:248–9.

18. Adzhubei I, Jordan DM, Sunyaev SR. Predicting Functional Effect of Human Missense Mutations Using PolyPhen‐2. CP Human Genetics. 2013;76.

19. Li M, Schroder R, Ni S, Madea B, Stoneking M. Extensive tissue-related and allele-related mtDNA heteroplasmy suggests positive selection for somatic mutations. Proc Natl Acad Sci U S A. 2015;112:2491–6.

20. Stoneking M. Hypervariable Sites in the mtDNA Control Region Are Mutational Hotspots. The American Journal of Human Genetics. 2000;67:1029–32.

21. Ramos A, Santos C, Mateiu L, Gonzalez Mdel M, Alvarez L, Azevedo L, et al. Frequency and pattern of heteroplasmy in the complete human mitochondrial genome. PLoS One. 2013;8:e74636.

22. Irwin JA, Saunier JL, Niederstatter H, Strouss KM, Sturk KA, Diegoli TM, et al. Investigation of heteroplasmy in the human mitochondrial DNA control region: a synthesis of observations from more than 5000 global population samples. J Mol Evol. 2009;68:516–27.

23. Soares P, Ermini L, Thomson N, Mormina M, Rito T, Rohl A, et al. Correcting for purifying selection: an improved human mitochondrial molecular clock. Am J Hum Genet. 2009;84:740–59.

24. Rebolledo-Jaramillo B, Su MS, Stoler N, McElhoe JA, Dickins B, Blankenberg D, et al. Maternal age effect and severe germ-line bottleneck in the inheritance of human mitochondrial DNA. Proc Natl Acad Sci U S A. 2014;111:15474–9.

25. Woerner AE, Cihlar JC, Smart U, Budowle B. Numt identification and removal with RtN! Bioinformatics. 2020;36:5115–6.

26. Marshall C, Parson W. Interpreting NUMTs in forensic genetics: Seeing the forest for the trees. Forensic Science International: Genetics. 2021;53:102497.

27. Strobl C, Eduardoff M, Bus MM, Allen M, Parson W. Evaluation of the precision ID whole MtDNA genome panel for forensic analyses. Forensic Sci Int Genet. 2018;35:21–5.

28. McElhoe JA, Holland MM, Makova KD, Su MS, Paul IM, Baker CH, et al. Development and assessment of an optimized next-generation DNA sequencing approach for the mtgenome using the Illumina MiSeq. Forensic Sci Int Genet. 2014;13:20–9.

29. Payne BA, Wilson IJ, Yu-Wai-Man P, Coxhead J, Deehan D, Horvath R, et al. Universal heteroplasmy of human mitochondrial DNA. Hum Mol Genet. 2013;22:384–90.

30. Samuels DC, Li C, Li B, Song Z, Torstenson E, Boyd Clay H, et al. Recurrent tissue-specific mtDNA mutations are common in humans. PLoS Genet. 2013;9:e1003929.

31. Just RS, Irwin JA, Parson W. Mitochondrial DNA heteroplasmy in the emerging field of massively parallel sequencing. Forensic Sci Int Genet. 2015;18:131–9.

32. Burr SP, Pezet M, Chinnery PF. Mitochondrial DNA Heteroplasmy and Purifying Selection in the Mammalian Female Germ Line. Dev Growth Differ. 2018;60:21–32.

33. Ye K, Lu J, Ma F, Keinan A, Gu Z. Extensive pathogenicity of mitochondrial heteroplasmy in healthy human individuals. Proc Natl Acad Sci U S A. 2014;111:10654–9.

34. Elliott HR, Samuels DC, Eden JA, Relton CL, Chinnery PF. Pathogenic mitochondrial DNA mutations are common in the general population. Am J Hum Genet. 2008;83:254–60.
